# Enhancing Career Interest Assessment in South Africa: Lessons Learned From the Development of the South African Career Interest Inventory-IsiXhosa Version

**DOI:** 10.3389/fpsyg.2022.854351

**Published:** 2022-07-11

**Authors:** Nicola Jansen van Vuuren, Stephan Rabie, Anthony Vernon Naidoo

**Affiliations:** ^1^Department of Psychology, Faculty of Arts and Social Sciences, Stellenbosch University, Stellenbosch, South Africa; ^2^HIV Mental Health Research Unit, Department of Psychiatry and Mental Health, Faculty of Health Sciences, University of Cape Town, Cape Town, South Africa

**Keywords:** South Africa, career interest inventory, assessment, interests, John Holland, language, culture

## Abstract

Career interest assessment is a vital component of career guidance and counseling. Ensuring fair and ethical assessment practice is, however, complex and challenging in a diverse multicultural setting such as South Africa. A myriad of factors—including culture, and language—may moderate individual performance on career interest assessments. For this reason, it is imperative to acknowledge these factors when developing career interest assessments in the South African context. Particular attention was paid to the issues of language and culture during the recent development of the South African Career Interest Inventory (SACII) and South African Career Interest Inventory-isiXhosa version (SACII-X). In this paper, we discuss some of the crucial considerations that informed the conceptualization and development of the SACII-X. The promising initial reliability and validity demonstrated by these assessments provide support for the use of career assessments based on imported career theories if sensitivity is afforded to contextual, language, and cultural considerations during the development of such assessment measures.

## Introduction

The assessment of career interests continues to be one of the most important constituents of career guidance and counseling (Harrington and Long, 2013). Traditionally, counselors and researchers in South Africa have used career interest measures directly imported from Europe and the United States of America. However, this practice has received increased criticism in recent years, and underscored the importance of using reliable, valid, and unbiased assessment measures to obtain accurate results that can be applied fairly. Morgan et al. (2021) aver that importing interest measures without evidence for their psychometric properties can be potentially problematic for several reasons. Imported interest measures might include items that do not adequately resonate with the local economic and cultural context (e.g., Einarsdóttir et al., 2002 cited in Morgan et al., 2021). These items can reduce the reliability of scale scores and lead to arbitrary response patterns. Furthermore, the underlying structural model of interests of these inventories might not be valid in the local context (see Leong and Pearce, 2014; Tao et al., 2019). This can impact the structural validity of the inventory and in a career counseling setting may potentially lead to inaccurate or misleading interpretations of interest scores (e.g., Tao et al., 2019). Rather than “throw the baby out with the bath water,” Morgan et al. (2021) argue that there is much to be gained by using a contextually relevant and normatively appropriate and valid interest measure in diverse cultural contexts. See Van de Vijver and Rothmann (2004) for a description of four kinds of procedures for dealing with multicultural assessment, namely: establishing equivalence of existing instruments, defining new norms, developing new instruments, and studying validity-threatening factors in multicultural assessment. In this paper, we focus on developing a new interest questionnaire in an indigenous South African language and engage with language and culture as potential threats to its validity.

Pertinent to this paper is John Holland’s (1985, 1997) theory of vocational personalities and work environments that has been widely used in career interest assessment (Nauta, 2010). Nevertheless, interest inventories used in South Africa based on Holland’s model such as the Self-Directed Search (SDS; Gevers et al., 1997) and the South African Vocational Interest Inventory (SAVII; Du Toit et al., 1993) contain outdated item content and questionable cross-cultural structural validity (Du Toit and De Bruin, 2002; Morgan, 2014).

Morgan et al. (2015a) developed the South African Career Interest Inventory (SACII) to address this shortcoming. However, the SACII was available initially only in English and Afrikaans, potentially excluding the majority of the South African population who are not English or Afrikaans home-language speakers. Rabie and Naidoo (2019) subsequently translated and adapted the SACII into isiXhosa to address this limitation, thereby developing the SACII-X, the first interest inventory in an indigenous South African language. Both these measures have shown promising reliability and validity in diverse South African samples (Morgan et al., 2015a; Rabie and Naidoo, 2019). The SACII and SACII-X have also been incorporated in career guidance interventions at high school level (see Naidoo et al., 2019 and Rabie et al. (2021) for an overview of the broader project). These findings provide support for the fair use of career interest assessments based on an imported model when tests are developed specifically for the cultural and language context of South Africa.

## Contextualizing the Development of the SACII and SACII-X

### Historical Influences on the Development and Use of Psychological Tests in South Africa

The topic of psychological assessment is contentious in South Africa, given its peculiar historical origins and development (Laher and Cockcroft, 2013) during the Apartheid era. The use of inappropriate and biased tests on race groups for whom they were not standardized was a common practice prior to and after the official legalization of apartheid (Laher and Cockroft, 2014; Foxcroft et al., 2018). Furthermore, during the Apartheid era, there were stark differences in career guidance services in the racially segregated schools (Watts, 1980). At White schools, these services were mandatory and well-resourced, whereas they received poor attention in schools for Black learners (Naidoo et al., 2017). This career guidance provision was informed by the extant job reservation apartheid policy with non-White students denied access to specific training and university-level studies—the preserve of White students.

During the 1980s and early 1990s, as apartheid laws and job reservation began to ease, the research and development of psychological assessments gained momentum (Laher and Cockroft, 2014). Nevertheless, due to the dearth of appropriate measures, assessment instruments were often imported and/or adapted from Western contexts (De Kock and Foxcroft, 2018), or measures developed and standardized for White South Africans were used in the assessment of other racial groups (Foxcroft et al., 2018). Given this history, it is crucial that the development and use of fair, reliable, and valid assessments are paid particular attention.

### Ethical Testing and Test Fairness

The history of psychological assessments in South Africa has partially informed legislation and guidelines for the ethical and fair use of psychological assessment instruments. The country’s new constitution in which basic human rights and equality of individuals are guaranteed has had a major impact on psychological assessment. This, according to Van de Vijver and Rothmann (2004), has shifted the spotlight to the demands on the cultural appropriateness of psychological tests and their usage with the promulgation of the new Employment Equity Act 55 of 1998, Section 8 (Republic of South Africa, 1998). This Act prescribes that all psychological measures be proven to be reliable and valid, unbiased against individuals or groups, and can be fairly used with an individual or group. Additionally, professional standards set by various professional bodies and the Health Professions Act (No. 56 of 1974; Health Professions Council of South Africa, 1974) prescribe that practitioners be conscious of any factors that may bias an assessment or moderate test performance, such as language and culture.

### Language and Culture as Moderators of Assessment Performance

Language is seen as one of the most notable moderators of psychological assessment performance, and thus a potential source of bias (Foxcroft and Aston, 2006). The insufficient understanding of words and concepts in a measure can result in differential item functioning (Foxcroft and Grieve, 2018a). This is a threat to the instrument’s reliability and validity, and the equivalence of test scores across individuals and groups (Abrahams and Mauer, 1999; Foxcroft et al., 2004). If test-takers are assessed in a language in which they lack adequate proficiency, their test results may not be an accurate reflection of their true standing on the construct (Foxcroft et al., 2004). Consequently, individuals may face discrimination based on their language proficiency, rather than their standing on a latent construct (Foxcroft et al., 2004). This highlights the importance of acknowledging language influences when evaluating the suitability of a career interest assessment, particularly in a multilingual context such as South Africa (Foxcroft and Aston, 2006).

Culture, closely intertwined with language, is an additional potential moderating factor in career interest assessment (Foxcroft et al., 2004). As such, imported Western career theories may not be suitable in the South African context as it may not adequately allow for the moderating effect of culture-specific beliefs and values on an individual’s career interests and decision-making processes (Albien and Naidoo, 2016). In addition, a test’s item content may reflect the culture of the test developers (Foxcroft et al., 2004; Foxcroft and Grieve, 2018a), regardless of the test’s language of administration (Van Eeden and Mantsha, 2007). Imported measures with irrelevant item content pose a threat to fair assessments (Foxcroft and Grieve, 2018a).

Accordingly, is it imperative that career interest tests underpinned by a Western model, such as Holland’s theory, have cross-cultural relevance in a multicultural society (Van Eeden and Mantsha, 2007). Simply translating a test may not be adequate—test adaptation to ensure applicability and relevance to the target culture is required (De Kock and Foxcroft, 2018). Within this context, the development of both the SACII and SACII-X holds promise in contributing to the research on the practice of career assessment and counseling in South Africa.

#### English as the Language of Instruction in South Africa

The choice of the most appropriate language of administration for career interest assessment in South Africa is a complicated issue. The *lingua franca* in South African business and education is primarily English (Foxcroft and Aston, 2006; De Kock et al., 2013; Desai, 2016). Hence, it could be argued that it is fair to assess test-takers in English, as inadequate proficiency in this language may deem the test-taker unsuitable for the workplace or tertiary education (Foxcroft and Aston, 2006). Some also argue that test-takers will be *disadvantaged* if they are assessed in a language different from their language of education as they would have acquired knowledge of words and concepts in English (McDonald and Van Eeden, 2014; Foxcroft and Grieve, 2018a).

Others such as Grobler and De Beer (2015) and De Kock and Foxcroft (2018) express concern about the assessment of individuals in a language other than their home language. This concern has been supported by research, indicating that non-native English speakers struggle to understand some of the English terminology used in, for example, the 16 Personality Factor Questionnaire (Abrahams and Mauer, 1999; McDonald and Van Eeden, 2014). Furthermore, despite English being the medium of instruction in many schools, it has been indicated that non-English home-language-speaking learners find this language of instruction challenging (Desai, 2016) and many learners are not adequately proficient in English upon completion of their schooling (Rabie and Naidoo, 2019). Assessing individuals in a language in which they are not adequately proficient may impact their score on a construct like career interests.

It can thus be argued that ethically test-takers should be able to complete assessments in their home language or language of choice (Foxcroft, 2011). This ensures the reliability and validity of test results, and the adherence to fair and ethical testing practices. Not only is this important from an ethical perspective, but also from a legal perspective, as it is unlawful according to the EEA in South Africa to use tests without evidence that it can be fairly applied to a test-taker and has been found to be reliable and valid.

Within the South African context, this implies that career interest measures should be developed and translated into more official languages, to provide test-takers with the opportunity (and the choice) to be assessed in their home language (Foxcroft et al., 2004; Albien and Naidoo, 2016).

### Validity of Imported Psychological Models: The Case of Holland’s Model

The validity of imported career interest models in the South African context forms another important consideration when developing career interest measures in South Africa (Van de Vijver and Rothmann, 2004). Holland’s RAISEC circular order model is a popular career interest model underpinning psychological instruments such as the SDS and the SAVII. The SDS has been shown to lack validity in the South African context. Du Toit and De Bruin (2002) found poor fit of Holland’s model as measured by the English SDS among a sample of over 1,400 Black South Africans with two indigenous African languages as their home language. Du Toit and De Bruin (2002) contend that test-takers experiencing difficulties in comprehending the meaning of the English items, as well as cultural differences in test-takers’ beliefs about careers and the meaning attached to Holland’s six interest types, may have contributed to the poor fit. Du Toit and De Bruin (2002) also mention socio-economic factors and outdated item content that does not align with South Africa’s current labor market as possible moderating factors on scores obtained on the SDS.

## Post-modern Approach to Career Assessment and Counseling

Several career researchers in South Africa (Maree, 2006; Stead and Watson, 2006; Alexander et al., 2010; Albien and Naidoo, 2018) have advocated for a post-modern career counseling discipline that needs to reflect innovative methods, techniques, and structures to ensure effective and culturally relevant career counseling. Furthermore, Bemath (2020) has critiqued the person-environment fit approach to career counseling, of which Holland’s model and the SDS form part, for lacking relevance in the South African context. This heralds a growing call for career counseling to shift from the trait-factor, objective approach to an interpretative process using new and creative qualitative ways of assessment. Maree and Beck (2004) aver for career counselors to be facilitators rather than prescriptive experts, and clients should be able to speak, act, think, and choose for themselves. Clients must be assisted to construct their own meaning in the career exploration process, and to take charge of their own choices and development.

In a similar vein, Morgan et al. (2019) contend that career counseling should not be a linear process with the counselor merely administering and scoring an interest inventory and then providing recommendations to the client based on these scores. Instead, career counseling should be a recursive process, in which both the counselor and client are actively engaged in constructing meaning (McIlveen and Patton, 2007; Maree, 2018), factoring in the realities of the client’s family and community contexts (Albien and Naidoo, 2017). The use of valid, reliable, and fair testing practices is a crucial component of this process. In a recent article in the aftermath of the COVID-19 pandemic, Maree (2022) recommends that a post-modern career construction approach can be used in rekindling hope and purpose in resource-constrained areas and help engage with inadequate “mastering of passive suffering” as well as interrupted mastering of developmental tasks during COVID-19. Maree (2018) suggests that the integrative strategy can be successfully implemented to help clients transition from career indecision; rediscover a sense of agency, hope, purpose, and meaning (Savickas, 2016); gain confidence in eliciting their career-life story; and draw on their own advice (under the guidance of a career counselor).

Several scholars have argued for the use of an integrated post-modern approach in career assessment and counseling that combines quantitative assessment with qualitative methodology (See Naidoo et al., 2017; Maree, 2022; Maree et al., 2022). In their qualitative + quantitative model, Maree et al. (2022) advocated that test scores are no longer used in isolation, but test scores and personal stories are integrated to promote critical self-reflection. Notwithstanding the merits of and the heavier emphasis on the qualitative component in this approach, the need for valid and culturally relevant quantitative interest measures is pertinent to this study.

## Challenges Related to Test Adaptation and Translation in South Africa

Considering the diversity of South Africa’s 11 official languages, adapting and translating career interest assessment measures into multiple language, is not devoid of challenges (De Kock and Foxcroft, 2018). Problems commonly encountered in the translation of measures include the absence of an equivalent term for a concept in the target culture, idiomatic expressions that cannot be translated directly, and the translation of the negative form in English that may confuse test-takers (De Kock and Foxcroft, 2018). In addition, the presence of different language dialects implies that familiarity with terms and expressions differs between subgroups speaking the same home language (Steele and Edwards, 2008). If test translators are not attuned to these nuances of a language, test translations may be inaccurate and could change the meaning and test-takers’ comprehension of items (Van Eeden and Mantsha, 2007).

Furthermore, as the language of instruction in South Africa is primarily English, the choice of the most suitable language for test administration is complex. The development of indigenous African languages has long been neglected, resulting in African languages being spoken, but not being developed as an academic or scientific language (Mda, 2004). Consequently, test-takers may choose to complete psychometric assessments in English, as this is their language of learning, rather than their home language.

## The Development of the SACII and SACII-X

The poor structural validity of imported career assessment measures based on Holland’s model among South African samples provided the impetus for the development of a theoretically and psychometrically based South African career interest inventory—the SACII. Morgan et al. (2015a) developed the SACII using a constrained emic approach, to ensure applicable item content. Items were written that are relevant to the emerging labor market of the country, as well as its unique language and cultural landscape (Morgan et al., 2015a).

Research on the SACII has shown promising results for the inventory’s reliability and validity across multilingual and multicultural South African samples. Nevertheless, the SACII was only available in English and Afrikaans, and the need existed to adapt and translate the inventory into isiXhosa, an indigenous African language, for inclusion in a high school career guidance project. Rabie and Naidoo (2019) subsequently developed the SACII-X, the first career interest inventory in an indigenous South African language. The translation of the SACII into isiXhosa involved both a judgmental, committee forward-translation design and a back-translation design (Rabie, 2017). The committee forward translations were done by isiXhosa home-language-speaking Masters’ students, one in Psychology and one in African languages, both fluent in isiXhosa and English. This collaborative translation ensured that no nuanced career psychology content was lost during the translation process, as well as ensuring a grammatically and linguistically correct translation (Rabie, 2017). In addition, the diversity of the South African isiXhosa-speaking population in terms of age, geographic location, and educational background was also considered in the adaptation and translation of the SACII (Rabie, 2017). As such, the translators paid careful attention to include language appropriate for the diversity of the isiXhosa-speaking population and the different sub-group dialects (Rabie, 2017). The challenges of the non-equivalence of certain isiXhosa and English terminology were addressed by directly translating and adapting items or terms into isiXhosa, and by presenting such terms in isiXhosa as a description of the meaning of the English concepts (Rabie, 2017). Hereafter, the SACII-X was back-translated by a bilingual, home-language isiXhosa-speaking Masters-level African Languages student. To determine the equivalence and accuracy of the isiXhosa translation, the original and back-translated versions of the SACII were compared. The second author is isiXhosa-speaking, with a degree in African Languages, and facilitated the adaptation and translation of the SACII-X using the Delphi-method.

Similarly to the SACII, the SACII-X has shown promising evidence in terms of the inventory’s reliability and structural validity in a sample of 266 isiXhosa home-language-speaking high school learners (Rabie and Naidoo, 2019). Additional South African studies have provided further support for both inventories’ psychometric properties (Morgan et al., 2015b, 2019; Mintram et al., 2019; Jansen van Vuuren, 2022). For instance, Mintram et al. (2019) found support for the fit and structural similarity of Holland’s model of vocational personality types for men and women, with slightly better fit reported for women. Similarly, Jansen van Vuuren (2022; Morgan, 2014) found support for the reliability and structural validity of the SACII-X, using the Rasch measurement model. Although sufficient model fit was obtained in Jansen van Vuuren’s (2022; Morgan, 2014) study, a small proportion of poor functioning items across the six SACII-X scales were observed. Recommendations regarding the adaptation or removal of the identified items were made, resulting in further improvement of the SACII-X.

The promising findings reported in the above-mentioned studies demonstrate the usefulness of Holland’s model as a depiction of vocational interest types in South Africa, if psychometric assessments that take the South African context into account are used. The development of the SACII and SACII-X has illustrated how test adaptation and translation difficulties as discussed in the previous section can be acknowledged and overcome. Furthermore, research such as Jansen van Vuuren’s (2022; Morgan, 2014) is crucial to provide psychometric corroboration for the initial findings and for improving existing career measures.

## Discussion and Conclusion

This paper highlights the considerations and challenges involved in developing a career interest assessment in the unique South African context and used the SACII-X as a case study in this pursuit. Career interest assessment is a cornerstone of career guidance practices (Harrington and Long, 2013). However, the indiscriminate use of invalid and unreliable career interest measures is unfair and potentially harmful to the career development of our clients.

It is imperative to acknowledge the historical influences that shaped the South African psychological assessment landscape, both from a social justice perspective and to ensure the implementation of ethical and fair testing practices. In addition, there exists clear evidence that language and culture can and do moderate assessment results. Crucially, through acknowledging the present linguistic and cultural diversity of the country, we are better placed to provide opportunities for culture fair psychometric assessment and informed career decision-making.

We acknowledge that the country’s eleven official languages and cultural diversity pose complex questions pertaining to the development of career interest assessments. However, as practitioners and researchers, we are professionally and morally obligated to apply our minds to this challenge and develop, research, and implement assessments that offer individuals from diverse backgrounds opportunities to receive fair testing and make informed career decisions. Even though the dominant language of instruction in South Africa is English, and many non-English home-language-speaking test-takers do choose to complete assessments in English (Foxcroft and Grieve, 2018b), test-takers should nevertheless be provided with the opportunity to complete assessments in their home language to promote test fairness and adhere to ethical guidelines.

The development of the SACII and SACII-X is recent example of how the challenges related to the complexities of the South African linguistic and cultural landscape can be acknowledged and addressed. It has provided an illustration of how a constrained emic approach to assessment development can successfully be applied in a diverse setting such as South Africa to ameliorate some of the concerns discussed, such as the impact of the country’s historical, socio-economic, cultural, and linguistic context may have on test results. Furthermore, it has shown the feasibility of a committee forward-translation and back-translation approach in translating measures. Including representatives of different cultural and linguistic subgroups in the translation process, provides another solution to ensure that translations account for the diversity of the South African language and cultural landscape. Through utilizing such procedures to develop, adapt, and translate career interest assessments in the South African context, we can provide individuals with equal opportunity to assess their career interests (Rabie, 2017).

The SACII and SACII-X have made a meaningful contribution toward the goal of reliable, valid, and fair career interest assessment in the South African context. The growing body of related research also holds implications for the cross-cultural structural validity of Holland’s model of vocational personality types as they build on previous findings that have found some support for the circumplex structure of Holland’s model in the African context (Morgan and De Bruin, 2018; Glosenberg et al., 2019; Morgan et al., 2021; Mashadza, 2020). This, in turn, holds important implications for the cross-cultural transportability of Holland’s model of vocational personality types into the African context.

However, we argue that these measures can serve as the catalyst for the development of other contextually and culturally relevant measures that represent other South African linguistic groups. Future research can consider comparing and analyzing English and isiXhosa results of the same cohort to assess item and method bias, and construct equivalence and other factors that can compromise the reliability of the instrument. A mixed-method design using a focus group may also ascertain high school participants’ perceptions of their preferences including those of the life orientation teachers.

While this study was necessarily situated in a quantitative design and utilized a relatively small sample in a peri-urban setting, more research is needed on how to use and apply the quantitative + qualitative narrative approach. Maree et al. (2022) and Rabie et al. (2021) have recently reported that such an integrated approach can also be applied effectively as a group intervention with participants from socially and economically disadvantaged contexts.

It is likely that there will be increasing numbers of members of various ethnic groups who will ask for culture-specific and culture-informed psychological practices in the coming years. Multicultural assessment is still in its infancy, but it is fair to assume that it will shift into sharper focus in the coming decades (Van de Vijver and Rothmann, 2004). It is in our own professional interest and ethical duty to our clients that we take care of it in an adequate way.

## Data Availability Statement

The raw data supporting the conclusions of this article will be made available by the authors, without undue reservation.

## Ethics Statement

The studies involving human participants were reviewed and approved by Stellenbosch Research Ethics Committee Stellenbosch University. Written informed consent to participate in this study was provided by the participants’ legal guardian/next of kin.

## Author Contributions

NJvV drafted major sections of the first version of the manuscript as part of her master’s dissertation. SR and AN were involved in the conceptualization and supervision of the study and contributed to specific sections and assisted with the final edits of the manuscript. All authors contributed to the article and approved the submitted version.

## Funding

Funding was provided by the Career Guidance High School Project, Departments of Psychology and Industrial Psychology, Stellenbosch University.

## Conflict of Interest

The authors declare that the research was conducted in the absence of any commercial or financial relationships that could be construed as a potential conflict of interest.

## Publisher’s Note

All claims expressed in this article are solely those of the authors and do not necessarily represent those of their affiliated organizations, or those of the publisher, the editors and the reviewers. Any product that may be evaluated in this article, or claim that may be made by its manufacturer, is not guaranteed or endorsed by the publisher.
